# Maternal history and uterine artery wave form in the prediction of early-onset and late-onset preeclampsia: A cohort study

**Published:** 2018-02

**Authors:** Nidhi Sharma, Krishnamurthy Jayashree, Kulasekaran Nadhamuni

**Affiliations:** 1 *Department of Obstetrics and Gynaecology, Saveetha Medical College, Chennai, India.*; 2 *Department of Radiology, Saveetha Medical College, Chennai, India.*

**Keywords:** Hypertension, Preeclampsia, Trophoblasts, Ultrasonography, Doppler, Uterine artery

## Abstract

**Background:**

Pregnancy induced hypertension (PIH) is a significant cause of maternal morbidity and mortality. Pregnancy-induced-hypertension can be prevented by identification of prenatal and antenatal factors. The uterine artery Doppler waveform transforms into a high flow with low resistance at 22-24 wk.

**Objective:**

To study the maternal risk factors and uterine artery Doppler waveform in singleton mid-trimester pregnancy and predict the occurrence of pregnancy-induced hypertension**.**

**Materials and Methods:**

This is a cohort study comprising of Doppler ultrasound examination of the uterine arteries at 20-23 wk gestation in 697 women with singleton pregnancies attending a routine target scan. The pregnant women were followed up. PIH was recorded in 57 (8.18%) of all pregnancies**.**

**Results:**

Maternal age >34 yr, primiparity, the presence of chronic hypertension was also associated with increased risk of PIH. High pulsatility index (>95^th^ percentile) as compared to low pulsatility index was a good tool for the detection of PIH (sensitivity 91.23% and specificity 99.06%, p<0.05). Presence of high pulsatility was a significant risk factor for early-onset PIH as compared to late-onset PIH.

**Conclusion:**

Uterine artery Doppler can be safely performed at the time of routine target anomaly scan in the second trimester. It is simple, economical, feasible and with good detection rates.

## Introduction

Pregnancy-induced hypertension affects 7-10% of all pregnancies and is a leading cause of maternal morbidity and mortality (1). The trophoblast associated remodeling of spiral arteries is completed by 14-16 wk (2). This is important because the maternal and the fetal circulations are not arranged in a formal counter current way (2). A significant fall in blood pressure must occur gradually from internal iliac artery to uterine artery and further into the radial and spiral arteries to keep the intervillous pressure low. The floating fetal tertiary stem villi in the intervillous space will collapse at high pressures leading to compression of villous capillaries and prevent maternal-fetal diffusion exchange (3). Though trophoblast does not invade the uterine artery directly, the uterine arterial blood flow is modified under the influence of vascular endothelial growth factor, placental growth factor and other angiopoietins released from the natural killer cells presented in the maternal decidua. These are secreted as early as 9 wk of gestation (4). 

Pulsatility Index can be defined as an objective assessment of uterine arterial waveform (Peak systolic flow velocity -End diastolic velocity/Mean velocity). The presence of early diastolic notch suggests increased vessel recoil and elasticity (low flow in early diastole). During normal pregnancy, the pulsatility index declines and the early diastolic notch should disappear latest by 24 wk. This happens because of the increased diastolic flow. Most prospective studies use a pulsatility index of >1.6 in the second trimester for the prediction of preeclampsia (5). The early diastolic notch can also be subjectively defined as a difference of 50cm/s from the maximum diastolic velocity after 20 wk (6). 

The uterine artery blood flow rises exponentially from 50 ml/min in a nonpregnant state to 600 ml/min during pregnancy (7) Recently, Magnetic resonance imaging usage in obstetrics with gadolinium definition of blood flow has demonstrated that most blood entering the uterine artery bypasses the spiral arterioles and intervillous space (8). Most blood entering the radial arteries traverse the uterus via the arteriovenous shunts and only a small proportion enters the intervillous space at a low pressure and velocity (9). The increased blood flow is much in excess of the local demand created by placental intervillous sinuses and provides to protect against maternal death from hemorrhage at delivery. 

As resistance increases in uterine arteries, the velocity of blood flowing into the intervillous space also increases from 10 cm/sec to 1-2 m/s and permanently damages the chorionic villi, leading to early-onset collapse of fetal capillaries in the stem villi (3). This results in reduced maternal-fetal diffusional exchange and early-onset preeclampsia. The ischemic reperfusion injury also generates oxidative stress (3). Hence this study was designed to study prospectively the risk of early- and late-onset preeclampsia in pregnancy with abnormal uterine artery waveform.

## Materials and methods

Doppler ultrasound examination of the uterine arteries was done at 20-23 wk gestation in women with singleton pregnancies attending a routine scan in second trimester. The pregnant women without fetal anomalies were offered the option of uterine artery Doppler evaluation for screening. The first-trimester scan finding was noted to date the pregnancy in all cases. 

This study was carried out on 697 pregnancies in the Department of Radiology and Department of Obstetrics and Gynecology at Saveetha Medical College and hospital, Chennai, India between 1 April 2015 and 31 December 2016. Multiple pregnancies and pregnancies with congenital anomalies were excluded. A detail maternal history was taken about parity, pre-pregnancy body mass index, previous low birth weight, hemoglobin levels, chronic hypertension, gestational diabetes and previous preeclampsia. A detailed examination of placenta was done sonographically. The ultrasonography equipment used was Philips HD11XE (Acuson, Mountain View, CA, USA); GE LOGICS7 Expert and Siemens Sonoline Acuson X150 (Siemens).

**Figure 1:**
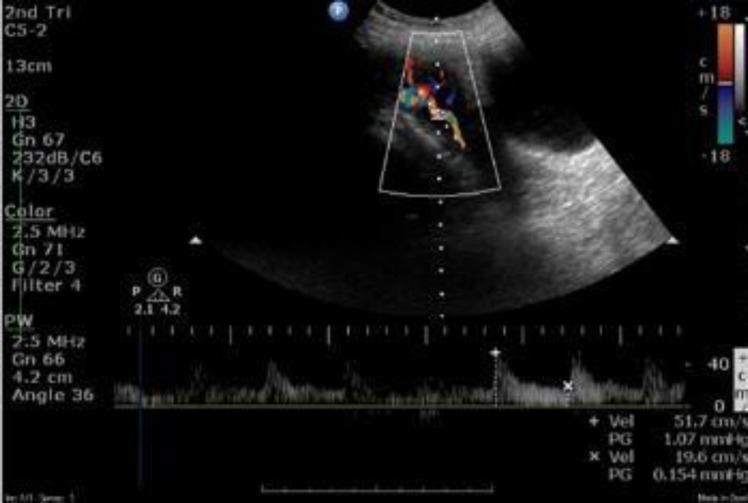
Doppler waveform of the uterine artery in the second trimester

The uterine artery was focused in the longitudinal scan lateral to the uterus. Pulsed wave Doppler was used to obtain three similar consecutive waveforms. The same was repeated for the contralateral uterine artery and the mean pulsatility index (maximum-minimum velocity/mean velocity) of the two vessels was calculated. The early diastolic notch was measured subjectively. The ultrasound transducer probe used was 3.5-or 5-MHz. It had spatial peak temporal average intensity of less than 100 mW/cm^2^. Waveform was measured in the absence of fetal breathing movements with fetal heart rate ranging from 120 -160 beats per minute. The angle of insonation of the ultrasound beam and the direction of blood flow was always kept less than 30 degrees (Figure 1).

Patients were called for antenatal visits every 2 wk till 36 wk and weekly thereafter. Blood pressure was measured in the right arm in the sitting posture and Koratkoff 5 sound as taken as the diastolic blood pressure. Urine albumin was measured at each visit. The guidelines of International Society for the Study of Hypertension in Pregnancy were used to define preeclampsia. Two recordings of diastolic blood pressure of ≥90 mmHg at least 4 hr apart in previously normotensive women were considered. A proteinuria of 300 mg or more in 24 hr, or two readings of at least + + on dipstick analysis of midstream or catheter urine specimens if no 24-hr collection is available was used to define proteinuria If hypertension and/or proteinuria developed before 34 wk the gestation was classified as early onset PIH. If hypertension and/or proteinuria developed after 34 wk the pregnancy was diagnosed as the late-onset PIH.

**Ethical consideration**

This study was approved by the ethical and research board (002/001/2016/SU/IEC). Written consent was obtained in all cases in the local language. 

**Statistical analysis**

Descriptive statistics was used to find significant levels of differences. Mean pulsatility index was not normally distributed in the frequency bar diagram in the study population and so it was expressed median ± percentile range. Fischer exact test and chi square test were used to analyze maternal history variables. The sensitivity, specificity, positive predictive value, negative predictive value, and likelihood ratio for a cut-off mean PI of 1.55 (95^th^ percentile) was calculated and bilateral or unilateral notches in the prediction of PIH were calculated. Differences were considered significant when p<0.05. Logistic regression was used to obtain the Odd’s ratio (OR) and 95% confidence interval (CI). Statistical analysis was done using Medcalc.

## Results

**Figure 2:**
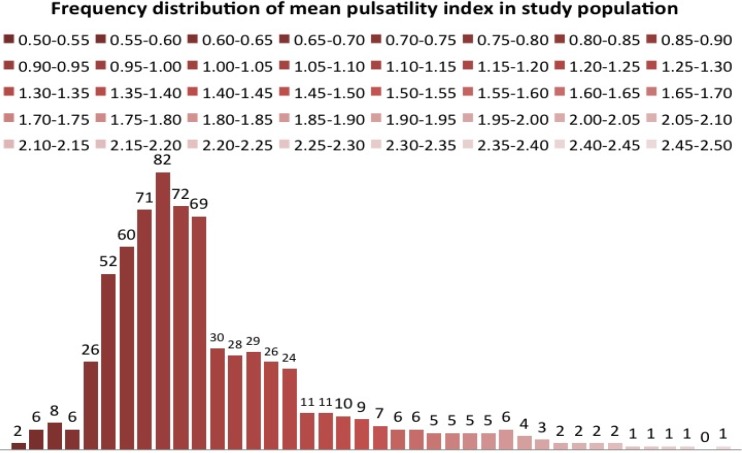
Frequency distribution of mean pulsatility index in the study population. Mean pulsatility index was not normally distributed and therefore expressed median±percentile range

Doppler examination was done in 750 pregnancies. Satisfactory waveforms were obtained in 743 pregnancies (99%). During the study period, a follow up was available for a total of 697 pregnancies. Uterine artery pulsatility index was not normally distributed but was found skewed to the right with the 95^th^ percentile at 1.55 (Figure 2). A total of 57 (8.18%) pregnancies resulted in pregnancy-induced hypertension out of total 697 pregnancies. There were no intrauterine deaths. 

Out of 57 hypertensive pregnancies, 25 were early onset (<34 wk) and 32 were late onset (>34 wk). There were 6 cases of grade 1 abruption. There were two cases of grade-three abruption. Out of 32 intrauterine growth restricted newborns, 31 survived beyond 4 wk of life.

Table I brings up the association of maternal history with PIH. There was a significant association between maternal age >34 and PIH (OR 3.3226, CI 1.1857-9.3110, Z statistic 2.284, p<0.0224). Primiparity had a significant association with pregnancy-induced hypertension (OR 2.2612, CI 1.3015-3.9286, Z statistic 2.895, p<0.0038). Presence of chronic hypertension was associated with a higher risk of preeclampsia (OR 23.8125, CI 7.6775-73.8571, Z statistic 5.489, p<0.0001). Both pre-gestational and gestational diabetes conferred a significant risk for the development of preeclampsia (OR 11.8125, CI 4.5813-30.4575, Z statistic 5.109, p<0.0001). This suggests that maternal history variables age, primiparity, chronic hypertension and diabetes mellitus are associated with an increased risk of developing PIH.

Table II brings up the fact that presence of high pulsatility is a significant risk factor for early-onset PIH as compared to late-onset PIH. Table III shows the sensitivity, specificity, positive predictive value, negative predictive value, likelihood ratio positive and likelihood ratio negative of early and late onset PIH when PI >1.55. This shows that an increased pulsatility index of >1.55 (95^th^ percentile) is associated with increased odds of developing PIH.

**Table I:** Screening characteristics of maternal history in pregnancy-induced hypertension

**Criterion**	**Characteristic**	**OR**	**Z statistic**	**CI**	**p-value (level of significance)**
Age	>34	3.226	2.284	1.1857-9.3110	0.0224
Nulliparity	Yes	2.2612	2.895	1.3015-3.9286	0.0038
Chronic hypertension	Yes	23.8125	5.489	7.6775-73.8571	<0.0001
Diabetes	Yes	11.8125	5.109	4.5813-30.4575	<0.0001

A p-value of less than 0.05 suggests a significant association of maternal history with pregnancy-induced hypertension.

OR: Odd’s ratio

CI: Confidence interval

**Table II:** Early- and late-onset pregnancy induced hypertension in high pulsatility and low pulsatility index

**Uterine artery PI**	**Early onset PIH**	**Late-onset PIH**	**Total PIH**
>1.55	24	28	52
<1.55	1	4	5
Total outcome	25	32	57

PI: Pulsatility index

PIH: Pregnancy induced hypertension

**Table III:** Screening characteristics mean pulsatility index (PI) > 1.55 and/or early diastolic notch

**Outcome**	**Sensitivity**	**Specificity**	**PPV%**	**NPV%**	**LR+**	**LR-**
PIH	91.23	99.06	89.66	99.22	97.31	0.09
PIH<34	96.00	94.44	41.38	99.84	18.97	0.04
PIH>34	87.50	95.49	48.28	99.37	19.40	0.13

PIH: Pregnancy induced hypertension

PPV: Positive predictive value

NPV: Negative predictive value

LR: Likelihood ratio

## Discussion

At the fetomaternal interphase, the first stage of remodeling of spiral arterioles, as early as 9 wk, is associated with vacuolations in the tunica media and intima of spiral arterioles under the influence of vascular endothelial growth factor, placental growth factor and angiopoiteins secreted from the maternal decidual natural killer cells (10). The second stage of remodelling is a proliferation of endovascular and interstitial trophoblast. In the second stage, the endovascular trophoblast actually blocks the lumen of spiral arteriole (11). 

In the third stage of remodelling there is loss of smooth muscle cells and elastic tissue from the vessel walls and this results in the dilatation of the opening of spiral artery into the intervillous space within the placenta. The remodelled arteries are not able to constrict their lumen due to absence of muscular layer, and thereby the blood supply to the placenta is continuous. Furthermore, the velocity and pressure of the inflowing maternal blood is significantly less and thus the hemodynamic damage to the delicate fetal tertiary stem villi is minimal. Finally in stage 4, there is re-endothelialization of spiral arterioles by maternal origin or trophoblast origin tissue, the origin of which is still being debated (11).

Impaired secretion of vascular endothelial growth factor, placental growth factor and angiopoietins from maternal decidua natural killer cells can lead to persistent protodiastolic notch and high downstream resistance, high-velocity diastolic flow (12). Impaired blocking of spiral arterioles by endovascular trophoblast can lead to high oxygen tension at fetomaternal interphase (13, 14). 

If there is impaired phagocytosis by trophoblasts this leads to retention of varying amounts of smooth muscle in the vessel walls. If smooth muscles are retained the opening of spiral arteries are narrow. The blood enters the intervillous space at a very high velocity in jet-like spurts. The perfusion is intermittent due to constriction of the vessel walls and diastolic flow is less. Defective remodelling can be associated with abnormal Doppler waveforms within the uterine arteries. The abnormality is usually elevated resistance in uterine arteries. The hemodynamic basis of impaired VEGF secretion and uterine artery high resistance flow is still to be determined (10). 

Screening characteristics of pulsatility Index are better for early-onset as compared to late-onset PIH (15, 16). These rheological studies suggest that early-onset PIH is an absolute noncompliant vascular system where the uterine artery waveform does not change in response to the angiopoiteins of pregnancy while late-onset PIH is a relative less compliant vascular system as happens in cases of increased maternal age, chronic hypertension or diabetes (18, 19). Association of high uterine pulsatility index and early onset PIH are supported by various other studies. It was found by Cnossen and colleagues that uterine artery Doppler ultrasonography can accurately predict pre-eclampsia. They concluded that the most powerful Doppler index for predicting pre-eclampsia is increased pulsatility index with early diastolic notching in the second trimester (6). 

The classification of PIH at or near term is clinical importance because early-onset PI is commonly associated with IUGR and iatrogenic preterm (21-23). The late-onset PIH is a milder form of maternal illness usually of renal origin and low rate of fetal involvement and the fetomaternal outcome is favorable (20, 22). Pregnant women with severe early-onset PIH also have different risk factors compared to late-onset PIH (24, 25). Our study shows that patients with early-onset PIH (<34 wk) have abnormal uterine Doppler mean PI, whereas, in late-onset PIH (>34 wk), only a proportion of these cases presented abnormal uterine Doppler assessment. This important finding suggests that there is a subgroup of late-onset cases with minimal placental involvement (23). Other studies have shown that early onset preeclampsia is a non-resilient cardiovascular system while late onset preeclampsia is probably renal in origin

Severe preeclampsia and early-onset preeclampsia is characterized by increased maternal and perinatal morbidity because of early onset PIH and prematurity. Thus, abnormal Doppler of the uterine artery may be considered as a local noninvasive imaging of a more generalized systemic vasculopathy. The high velocity, low volume, intermittent perfusion by the uterine artery supplying the intervillous space at the trophoblast-maternal interphase can cause hemodynamic and oxidative damage resulting in the maternal syndrome of PIH. The resulting ischemic reperfusion injury can lead to oxidative stress and PIH.

## Conclusion

The study was performed in maternity ultrasound clinics providing routine antenatal care rather than specialized medical personnel in fetal medicine units. Preeclampsia is a grave maternal complication and has an enigmatic pathophysiology. There are no established biomarkers and no standardized thresh holds. This unconventional approach shows that maternal history and Doppler ultrasound assessment of uterine arteries can detect PIH in its preclinical stage and timely intervention can help improve the fetomaternal outcome. The sensitivity and negative predictive value are better for early-onset PIH. Thus early onset preeclampsia is probably a disease of non-resilient cardiovascular system. The use of a uterine artery screening programme in routine antenatal care would help to identify pregnancies requiring further intense feto maternal surveillance. Uterine artery Doppler is safe at the time of routine target anomaly ultrasound scan in the second trimester. It is also economical and easy with good sensitivity.
